# Effectiveness of Manual Toothbrushing Techniques on Plaque and Gingivitis: A Systematic Review

**DOI:** 10.3290/j.ohpd.a45354

**Published:** 2020-10-02

**Authors:** Andrea Rani Rajwani, Sophia Nancy Diana Hawes, Amanda To, Alessandro Quaranta, Julio C. Rincon Aguilar

**Affiliations:** a Dentist, UWA Dental School, The University of Western Australia, Perth, Australia. Idea, experimental design, performed review, wrote manuscript.; b Dentist, UWA Dental School, The University of Western Australia, Perth, Australia. Idea, performed search, experimental design, performed review, wrote manuscript.; c Senior Clinical Specialist, Sydney Dental Hospital, Sydney, Australia; Scientific and Education Director, Smile Specialists Suite, Newcastle, NSW, Australia. Idea, experimental design, performed review, wrote manuscript.; d Senior Lecturer in Periodontology, UWA Dental School, The University of Western Australia, Perth, Australia. Idea, experimental design, performed review, wrote manuscript, proofread the manuscript.

**Keywords:** evidence-based medicine, oral hygiene, systematic review, toothbrushing

## Abstract

**Purpose:**

Currently, there is no consensus on recommendations for manual toothbrushing techniques between dentists, oral health therapists and dental companies. The aim of this systematic review is to identify and assess the quality of evidence of the effectiveness of manual toothbrushing techniques in the existing literature.

**Methods:**

A broad search was conducted on the electronic databases Medline via Ovid, PubMed and EBSCO Dentistry & Oral Sciences. Included studies examined manual toothbrushing technique efficiency. Articles were assessed utilising the Cochrane Collaboration’s tool for assessing risk of bias. Thirteen studies met the inclusion criteria and were included in this review. These included five randomised controlled trials (RCT), seven experimental non-randomised control studies and one in vitro study.

**Results:**

Of the 3190 articles identified, 40 were relevant to manual toothbrushing and 13 were included in the final review. Studies indicating statistically significantly superior plaque removal for a given technique were Bass (one), modified Bass (one), Charter’s (two), Fones (two), scrub (two), roll (one), modified Stillman (one), toothpick method (one). Four studies exhibited no statistically significant difference in effectiveness of plaque removal. Unfortunately, considerable variation was found between studies, making a definitive conclusion impossible in terms of an ideal manual toothbrushing technique that would promote plaque removal and reduce gingivitis.

**Conclusion:**

There is still insufficient evidence for suggesting that one toothbrushing method is more effective than another in plaque removal and reduction of gingivitis. Excessive variability in many aspects of the design and methodology of the selected studies hinder conclusions on an ideal manual toothbrushing technique. Experimental randomised controlled trials that follow the CONSORT guidelines are required to provide adequate-quality evidence and make any definitive conclusions on the relative effectiveness of manual toothbrushing techniques.

Improved oral hygiene remains a fundamental issue in dentistry for the prevention of oral disease. including dental caries, gingivitis and periodontitis. The initial stages of periodontal disease manifest as gingivitis,^32^ followed by irreversible bone loss and soft tissue attachment recession associated with numerous local and systemic effects, including tooth loss.^20,38^

Effective regular removal of the bacterial plaque with toothbrushing is the primary method for dental caries and periodontal disease prevention, and ceasing disease progression.^2,3^

Despite the introduction of electric toothbrushes and their increased affordability, manual toothbrushes are more frequently used in the United States^49^ and although no data are available for Australia, sales statistics indicate 35 million manual toothbrushes being sold annually, comprising of 80% of toothbrush sales in Australia.^43^ This suggests that manual toothbrushing remains prevalent. Therefore, manual toothbrushing techniques should continue to play a large role in patient oral hygiene education.

Numerous toothbrushing techniques have been developed over the last century, including Charter’s, Stillman, modified Stillman, Fones, Bass, modified Bass, scrub, roll and toothpick. Research has reinforced a greater improvement in plaque removal by educating patients in a specific technique as opposed to suggesting modifications to their existing technique.^14^ This further reinforces the need for defined and evidenced-based techniques.

The earliest published toothbrushing technique is the Charter’s method, which was originally described for patients with orthodontic appliances in 1928.^25^ The Stillman technique was developed by Paul R. Stillman^50^ in 1932 and involves the placement of toothbrush bristles partly on the cervical portion of the tooth and partly on the adjacent gingiva at a 45-degree angle. The modified Stillman technique involves the addition of a roll technique by brushing the final stroke towards the biting or occlusal surface. In 1934, Alfred Fones proposed a new eponymous brushing technique.^6^ In this technique, the toothbrush is placed on a set of teeth and the bristles used by slightly pressing them onto the interface between the tooth and the gingival margin. Then the toothbrush head is moved circularly 4 to 5 times. Then the toothbrush is placed on the next set of teeth. The Bass toothbrushing technique was first described by Charles C. Bass.^5^ Bass recommended forcing the toothbrush bristles into the gingival crevices and sulcus between teeth at a 45-degree angle to the long axis of the tooth with a ‘short back and forth movement’ of the brush to dislodge all soft material. The incorporation of a rolling action is known as the modified Bass technique. The modified Bass technique is still widely used today by general dentists and periodontists for patient education.^57^ The scrub technique is reported to be the least technique sensitive and has been described with either circular, horizontal or vertical strokes.^12,46^ The toothbrush head is placed at a 90-degree angle to the surface with either horizontal/vertical or circular motions used on the gingival crevice.

The roll technique is performed by placing the bristles parallel to the attached gingiva and executing repeated strokes towards the occlusal or incisal surface. This sweeping motion is conducted at a 90-degree angle to the tooth surface.^4^ First described in 1984, the toothpick method applies the toothbrush head at a 30-degree angle towards the crown and it is thrust between the interproximal areas of teeth eight to nine times.^58^

Currently, there is no consensus on recommendations for manual toothbrushing techniques between dentists, oral health therapists and dental product companies.^57^ It is important to define toothbrushing techniques and to determine which techniques are more effective in removing plaque in order to provide consistent and evidence-based oral hygiene education. The aim of this systematic review is to identify and assess the quality of evidence in the existing literature on the effectiveness of manual toothbrushing techniques in plaque removal and preventing gingivitis.

## Materials and Methods

A broad search was conducted in May 2018 utilising the electronic databases Medline via Ovid (Medline Ovid, 2018 National Library of Medicine, Bethesda, MD, USA), PubMed (PubMed, 2018 National Library of Medicine) and EBSCO Dentistry & Oral Sciences Source (EBSCO [Elton B. Stephens Co], 2018 Dentistry & Oral Sciences Source, Ipswich, MA, USA) to capture all published studies related to manual toothbrushing techniques. Databases were searched from the earliest year available on each database up to May 2018. Thirty-six search terms were used in total. These included ‘toothbrushing manual’, ‘brushing manual’, ‘Stillman’, ‘Stillman modified’, ‘Bass’, ‘Bass modified’, ‘Fones’, ‘Scrub’ and ‘Charter’s searched with the terms ‘technique’, ‘plaque’, ‘efficiency’ and ‘effectiveness’ (Table 1). Hand searching was also performed; the references of relevant papers were checked for any further studies. Articles corresponding with the search terms were identified; subsequently, titles and abstracts were screened for relevance.

**Table 1:** Search terms

Search terms	PubMed	Medline	DOSS
Toothbrushing manual AND technique	41	0	11
Toothbrushing manual AND plaque	425	3	42
Toothbrushing manual AND efficiency	28	0	1
Toothbrushing manual AND effecEveness	107	0	0

Brushing manual AND technique	49	0	9
Brushing manual AND plaque	465	1	40
Brushing manual AND efficiency	32	0	3
Brushing manual AND effecEveness	122	0	0

Stillman AND technique	72	3	6
Stillman AND plaque	15	4	2
Stillman AND effciency	23	0	0
Stillman AND effecEveness	27	2	0

Stillman modiﬁed AND technique	3	0	3
Stillman modiﬁed AND plaque	3	0	2
Stillman modiﬁed AND effciency	5	0	0
Stillman modiﬁed AND effecEveness	4	0	0

Bass AND technique	431	186	61
Bass AND plaque	84	71	73
Bass AND effciency	163	98	3
Bass AND effecEveness	259	55	0

Bass modiﬁed AND technique	51	0	30
Bass modiﬁed AND plaque	32	0	24
Bass modiﬁed AND effciency	7	0	2
Bass modiﬁed AND effecEveness	17	0	0

Fones AND technique	10	6	12
Fones AND plaque	6	7	6
Fones AND efficiency	3	0	0
Fones AND effectiveness	6	5	0

Scrub AND technique	20	325	13
Scrub AND plaque	27	27	6
Scrub AND efficiency	27	74	0
Scrub AND effectiveness	110	0	0

Charters AND technique	0	0	0
Charters AND plaque	3	0	0
Charters AND efficiency	0	0	0
Charters AND effectiveness	0	0	0

**Fig 1:**
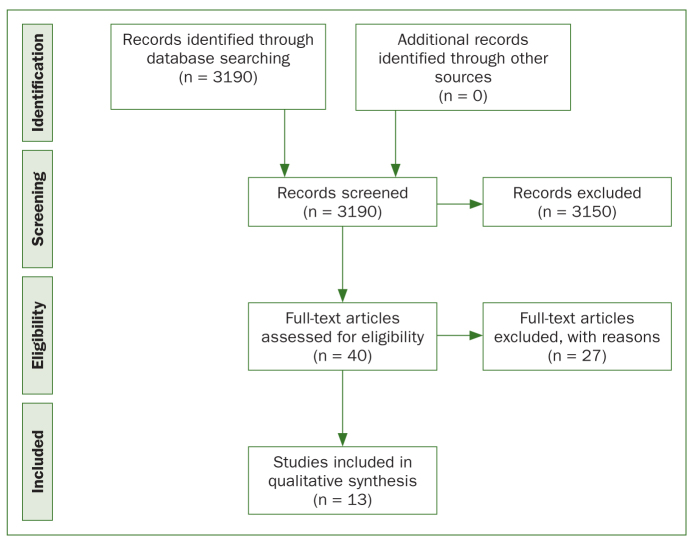
Flowchart of systematic review process.

Following screening, the remaining articles were blinded for title, authors, and publication details including journal and any author affiliations. Two researchers (SH, AT) independently conducted the literature search and independently graded each article utilising the Cochrane Collaboration’s tool for assessing risk of bias.^22^ This tool was used to ensure a systematic and explicit approach to assess the quality of evidence of the studies. Any disagreement on the inclusion or exclusion of articles or on grading was resolved by discussion and consensus. The study design is shown utilising the Preferred Reporting Items for Systematic Reviews and Meta-Analyses (PRISMA [Preferred Reporting Items for Systematic Reviews and Meta-Analyses])^34^ flow diagram (Fig 1). The inclusion and exclusion criteria for the research are described in Table 2.

**Table 2:** Inclusion and exclusion criteria

Inclusion criteria	Exclusion criteria
**P: Participants** Participant age 17 years or older, any gender.	**P: Participants** Younger than age 17, compromised manual dexterity, orthodontic appliances ﬁxed, removable prosthesis.
**I: Interventions** Manual toothbrushing techniques, Charter’s modiﬁed Bass, Bass, Stilllman, modiﬁed Stillman, Fones, Scrub.	**I: Interventions** Electric toothbrushing or electric toothbrushes, types of toothbrush, interdental cleaning, mouthrinse, modiﬁed toothbrush-holding techniques.
**C: Comparisons** Baseline plaque scores or bleeding on probing, no cleaning, ﬂossing only.	**C: Comparisons** Study design: reviews, case reports, abstracts, letters to the editor, commentaries, animal studies. Language: non-English language studies excluded. Full-text article not available.
**O: Outcomes** Changes in oral health by comparison to baseline measured in plaque scores, BOP scores. Publication year: First available year of each database. Study designs: randomised control trials, split mouth, observational, in vitro.	

Focused question: What is the efficacy of manual toothbrushing techniques on pre- and post-instruction scores on bacterial plaque control and gingivitis?

## Results

### Literature Search and Excluded Studies

A wide search of the literature was conducted in May 2018, identifying 3190 articles. Most of these articles were either duplicates or involved only electric toothbrushes or comparisons of toothbrush design (e.g. type of toothbrush head). Following screening, 40 articles related to manual toothbrushing techniques were identified and read in full-text; 27 articles were excluded.

Two review articles were excluded.^47,57^ Eight articles were excluded upon further examination due to their intervention protocols, as they investigated different toothbrush designs only (no technique),^8,42^ force and duration of toothbrushing,^14,55^ ability of participants to adopt a new toothbrushing method,^44^ biofilm models in mechanical plaque removal,^53^ comparisons of left and right handers^28^ or brushing sequence.^52^

Four studies were excluded due to their study sample: one was an animal study on monkeys,^56^ two included participants with orthodontic appliances^31,36^ and one with removable prostheses.^10^ Six studies^16,27,37,41,48,51^ involving participants under 17 years were excluded, as were seven articles due to lack of an English version.^11,17,21,23,30,40,59^

Following the exclusion of 27 studies, 13 studies were included in the final review (Fig 1).

### Articles Included in the Study

Thirteen studies met the inclusion criteria and were included in this review (Table 3). These include five randomised controlled trials (RCT),^1,18,19,33,45^ seven experimental non-randomised control studies^7,12,13,15,29,35,39^ and one in vitro study.^24^ In these 13 studies, ten manual toothbrushing techniques were examined with the number of studies indicated in parenthesis: roll (six), horizontal scrub (six), modified Bass (five), Bass (five), Charter’s (three), Fones (three), modified Stillman (one), toothpick method (one), and vertical method (one). The results section outlines the study design and the findings of the study from each technique.

**Table 3:** Studies, toothbrushing techniques, plaque reduction scores and results

Toothbrushing techniques publications	Charter’s	Scrub (horizontal)	Roll	Modified Stillman	Bass	Electric	Fones	Modified Bass	Circular scrub	Toothpick	Vertical	Result
Frandsen et al 1970^12^	A 1.12 (62%) B 0.9 (49%)	A 1.04 (58%) B 1.18 (66%)	A 0.89 (47%) B 0.82 (48%)									Charters better than roll or scrub for brusher a, scrub was better for brusher b
Frandsen et al 1972^13^	A 1.25 (75%) B 1.30 (77%) C 1.41 (83%)	A 1.13 (66%) B 1.34 (79%) C 1.24 (74%)	A 1.20 (72%) B 1.04 (63%) C 1.08 (64%)									Pending on instructor Charter’s & scrub more effective
Arai and Kinishita 1977^1^	Plaque score see paper 58.1+16.3	Plaque score see paper 71.7 +11.1%	Plaque score see paper 62.9+13.6%	Plaque score see paper 67.4+13.7%	Plaque score see paper 55.2+10.2	Plaque score see paper 64.7+13.7%	Plaque score see paper 75.2 +9.9%					Fones and scrub more effective
Gibson and Wade 1977^15^			1243 (54.52%) 1184 (51.93%)		1199 (52.59%) 1234 (54.12%)							No difference between techniques
Bergenholtz et al 1984^7^		Index A 5.6 V 6.5 S Index B see publication	Index A 5.7 V 6.5 S Index B see publication		Index A 5.2 V 6.0 S Index B see publication				Index A 5.3 V 5.9 S Index B see publication			No difference between toothbrushes, Bass better and roll less effective
Kanchanakamol et al 1993^29^			MNPI 1.09		MNPI 1.4							Bass technique with tooth brush concept 45 best mean plaque reduction MNPI
Morita et al 1998^35^					Supervised prox 1.34 (38.8+9.4%) Bucco-linugal 0.95 (55.7+14.0%) Non-supervised prox 1.33 (41.6+14.1%) Bucco-linugal 0.84 (61.9+19.6%)					Supervised prox 0.97(57.5+12.5%) Bucco-linugal 0.95 (58.3+10.5%) Non-supervised prox 0.99(57.8+13.9%) Bucco-linugal 0.92 (59.9+14.5%)		Toothpick method better than Bass
Poyato-Ferrera et al 2003^39^								Normal toothbrush vs M-Bass TQHI Normal M-Bass D2 0.42-2.46 0.28-2.94 D7 0.12-2.26 0.21-2.00 D21 0.23-2.60 0.01-1.62				Modified Bass statistically significantly better than normal
Harnacke et al 2012^18^							See publication graphs for PBI, TQHI AND MPI	See publication graphs for PBI, TQHI AND MPI				Fones superior to bass for both OH skills and gingivitis
Schlueter et al 2013^45^								Normal toothbrush vs M- Bass TQHI Baseline No instruction 1.99 +0.51 Leaflet 1.90 +0.51 Demo 1.93+0.56 - Post-intevention No instruc 1.80+0.47 Leaflet 1.58 +0.58 Demo 1.64+0.58				No significant differences between all groups
Mastroberardino et al 2014^33^		RMNPI 0.40+0.10 32.20%									RMNPI 0.37+0.09 36.20%	Vertical significantly better overall plaque removal than horizontal and better interdental
Harnacke et al 2016^19^							MPI 70.3% + 14.7 BOP no difference	MPI 77.91 + 14.37 BOP no difference				Fones with computer training better than modified Bass for oral hygiene. No differences for gingivitis
Jansiriwattana et al 2018^24^		O’Leary Butler 70.88 Colgate 70.02 ORAL B 68.56 PMI B L Butler 0.00 1.15 Colgate 0.05 1.00 Oral B 0.02 1.17						O’leary Butler 67.64 Colgate 67.48 Oral B 67.80 PMI B L Butler 0.05 0.88 Colgate 0.21 1.21 Oral B 0.03 1.18				No difference between toothbrushes or techniques

### Toothbrushing Techniques

We were able to identify 10 different manual toothbrushing techniques compared in the thirteen selected studies. Nine studies compared only two manual brushing techniques.^15,18,19,24,29,33,35,39,45^ Out of these nine studies, five compared modified Bass technique vs other techniques.^18,19,24,39,45^ One compared horizontal vs vertical toothbrushing techniques.^33^ Two studies compared the Bass technique to the roll technique.^15,29^ Morita et al^35^ compared the Bass vs the toothpick technique.

Two studies by Frandsen et al^12,13^ compared three manual toothbrushing techniques: Charter’s vs scrub vs roll. Bergenholtz et al^7^ compared four different toothbrushing techniques: scrub vs roll vs Bass vs circular scrub. Finally, Arai and Kinishita^1^ compared seven different toothbrushing techniques, six with manual toothbrushes and one with electric: Charter’s vs scrub vs roll vs modified Stillman vs Bass vs Fones vs electric (Table 3).

### Use of Bacterial Plaque Reduction and Bacterial Plaque Indices

The use of percentage of bacterial reduction in the studies included in this systematic review was described in four studies.^1,12,13,15^ Two studies reported reduction of bacterial plaque by using mean plaque reduction.^33,35^ Seven studies provided no information about percentage of bacterial plaque reduction (Table 3).^7,18,19,24,29,39,45^

Ten different plaque index systems were used in the thirteen studies included in this review. Arai and Kinoshita^1^ used a Modified Volpe’s Plaque Score. Frandsen et al^12,13^ used the Silness and Löe bacterial plaque score. Mastroberardino et al^33^ used the Rustogi Modified Navy Plaque Index (RMNPI). Kanchanakamol et al^29^ used the Modified Navy Plaque Index (MNPI). Bergenholtz et al^7^ used a method he developed, which is a modification of the Silness and Löe plaque score. Gibson and Wade^15^ used the Podshadley and Haley plaque index. Jansiriwattana and Teparat-Burana^24^ used two indices, the O’Leary plaque index and the Proximal Marginal Index (PMI) by Benson. Harnacke et al^18^ used two indices in their first publication, the Marginal Plaque Index (MPI) by Deinzer et al^9^ and the Turesky modification of the plaque index of Quigley & Hein (TQHI). A second study by Harnacke et al^19^ just used the MPI by Deinzer et al.^9^ Four studies used the TQHI index (Table 4).^18,35,39,45^

**Table 4:** Publications and variables

Publications	Plaque score index used	Number and type of manual toothbrushes	Participants’ age, background and number	Type of brushing training and methodology	Gingival index used	Teeth and surfaces assessed
Frandsen et al 1970^12^	PI Silness and Loe	One manual toothbrush, Lactona m 39 nylon	US Army 17-25 years n=60	Left side brushed by a dentist, brusher A Right side by a hygienist, brusher B	None	All teeth 4 surfaces
Frandsen et al 1972^13^	PI Silness and Loe	One manual toothbrush, Lactona m 39 nylon	University students, 18-27 years, n=182	Seven instructions, three instructors Teaching all 3 methods a, b & c	None	All teeth 4 surfaces
Arai and Kinishita 1977^1^	PI Modified Volpe’s	Nine types of toothbrushes 7 Manual, 2 electric, see document for brands	Adults, dentist, dental students, hygienists 20-34 years, n=52	Examiner monitoring dental trained participants	None	Six teeth Ramfjord teeth
Gibson and Wade 1977^15^	PI Podshadley and Haley	Four manual toothbrushes: Oral-B 40, Sensodyne Softex, Wisdom nylon medium, Wisdom multi-tuft, short head	Dental students, no age description, n=38	Participants trained by hygienist: verbal, model and intra-oral demo, tests	None	Six teeth Ramfjord teeth
Bergenholtz et al 1984^7^	Bergenholtz index	Two types of manual toothbrushes, v-shaped vs straight, Jordan A/S brand	Previous university perio patients 20-49 years, n=24	Patients brushing, part A Trained dental assistants brushing, part B	None	Index a All teeth 4 surfaces Index b All teeth 10 surfaces
Kanchanakamol et al 1993^29^	PI Modified Navy	Two manual tooth brushes, conventional “Concept 45”	Soldiers at an airbase, 20-21 years, n=100	Trained in a small group, 2-hour session	None	All teeth 8 surfaces full dentition
Morita et al 1998^35^	PI Turesky Modfied Quigley & Hein	Manual toothbrush with 2 rows of nylon bristles of 6 tufts per row and 50 filaments per tuft, no brand described	Male university dental students, 20-26 years, n=20	Teeth brushed by examiner exp 1, participants brushed own teeth after instruction, exp 2	None	TQHI all teeth six surfaces
Poyato-Ferrera et al 2003^39^	PI Turesky Modfied Quigley & Hein	Manual tooth brush Vitis Dentaid	Secondary students, 18-30 years, n=46	Part 1 normal home brushing, part 2 trained using a model video	None	TQHI all teeth six surfaces
Harnacke et al 2012^18^	Turesky modfied Quigley & Hein Marginal Plaque Index Deinzer et al	Manual tooth brush Elmex Inter X, Gaba	University students (not dental students), average age c 23.5 f 23.2 b 22.9, n= 67	Written text, slides and video presentation of each technique	Papillary bleeding index for gingivitis	MPI all teeth eight surfaces TQHI all teeth six surfaces
Schlueter et al 2013^45^	PI Turesky Modfied Quigley & Hein	Manual tooth brush Elmex Inter X, Gaba	University students (not dental students), mean age 26.6 years, n=98	Control: no instruction Leaflet instruction Demonstrations using a tooth model	None	TQHI all teeth six surfaces
Mastroberardino et al 2014^33^	Rustogi Modified Navy Plaque Index	Manual toothbrush Mentadent Tecnic Clean	University students (not dental students), age 19-24 years, n=61	Brushing by trained dental hygienist	None	All teeth 18 areas
Harnacke et al 2016^19^	Marginal Plaque Index Deinzer et al	Manual tooth brush Elmex Inter X, Gaba	Random adults, age 22-23 years, n=70	Computer slides with training instructions of Fones vs M-Bass	Bleeding on probing (BOP)	MPI all teeth eight surfaces
Jansiriwattana et al 2018^24^	O’Leary plaque index, proximal marginal index (PMI) by Benson	Three manual tooth brushes, Colgate 360 Oral B Pro Health Butler Gum 311	11 tests per 3 manual toothbrushes, x2 Techniques total 66 tests	In vitro study, toothbrushing by a calibrated dentist	None	Six surfaces all teeth in vitro models

### Number and Type of Toothbrushes

In terms of the types of toothbrushes used, the 13 studies contained multiple variations. Eight studies compared different toothbrushing techniques using one type of manual toothbrush for all study participants.^12,13,18,19,33,35,39,45^ Two studies compared toothbrushing techniques using two different types of manual toothbrushes.^7,29^ In an vitro study, Jansiriwattana and Teparat-Burana^24^ compared two toothbrushing techniques using three different manual toothbrushes. Gibson and Wade^15^ used four different types of manual toothbrushes. In 1977, Arai and Kinoshita^1^ used seven manual and two electrical toothbrushes. Counting the total number of toothbrushes in all thirteen studies, 23 different types of manual toothbrushes were used (Table 4).

### Participant Age, Background and Numbers

Eleven studies recruited individuals between 17 and 34 years old,^1,12,13,15,18,19,29,33,35,39,45^ and only one study recruited previous periodontal patients aged 20 to 49 years old.^7^ One study was in vitro, using dental plastic models with 28 teeth.^24^

Most of the studies recruited university students;^1,13,15,18,33,35,45^ out of these seven studies, three included dental students or dental related staff.^1,15,35^ Two studies recruited young members of the armed forces aged 17 to 25.^12,29^ One study recruited highschool students^39^ and another study enrolled young random adults 22 to 23 years old from a German town.^19^ Only one study enrolled previously periodontologically treated patients from a university clinic.^7^

Four studies recruited between 20 and 50 individuals.^7,15,35,39^ Eight studies recruited between 51 to 100 individuals.^1,12,18,19,29,33,45^ Only one study recruited more than 100 individuals (Table 4).^13^

### Toothbrushing Training Methodology

Oral hygiene instructions and training were delivered to participants using different methodologies. One study organised a 2-h training session for small groups of soldiers at an airbase.^29^ In the study by Arai and Kinoshita,^1^ a dental examiner supervised dental students, dentists and hygienists in performing toothbrushing techniques properly. Five studies used trained dentists, dental hygienists or dental assistants and they brushed participant’s teeth.^7,12,24,33,35^ Six studies used written, computer, video, model and verbal instructions to let participants use the technique (Table 4).^13,15,18,19,39,45^

### Comparison of Bacterial Plaque Reduction

The following results from each publication are described in terms of bacterial plaque control.

Frandsen et al^12^ reported results as reduction of mean plaque scores and percentage reduction of bacterial plaque. Charter’s was better than the roll technique for brusher A and the scrub technique was better than than Charter’s for brusher B.

In a second study by Frandsen et al,^13^ results were similarly reported and no significant differences were found between roll, scrub and Charter’s techniques. Charter’s and scrub were slightly more effective in removing plaque as per reduction percentage of plaque scores.

Arai and Kinoshita^1^ reported results as the average percentage of plaque removal. They found the Fones and Scrub methods to be more effective than the other manual toothbrushing techniques. Gibson and Wade^15^ presented results as total plaque scores and percentage areas exhibiting plaque. They found no statistically significant differences between the roll and Bass techniques.

Bergenholtz et al^7^ presented their results comparing toothbrushing techniques by the use of Index A and Index B. Index A showed more plaque removal ability for the Bass technique with scores of 5.2 for V-shaped toothbrushes and 6.0 for S toothbrushes. That study also aimed to compare straight (S) and V-shaped toothbrushes. They reported no difference in plaque removal ability (Index B) on buccal and lingual surfaces when professionally cleaned by trained dental assistants.

Kanchanakamol et al^29^ reported better mean reduction according to the MNPI for the Bass technique compared to the roll technique using the ‘Concept 45’ toothbrush. Morita et al^35^ compared the Bass and toothpick methods by the mean plaque index and mean percentage plaque reduction. The toothpick method removed more plaque than did the Bass method.

Poyato-Ferrera et al^39^ compared normal toothbrushing practises with the Modified Bass technique using the TQHI. The modified Bass plaque technique was statistically significantly more effective in removing supragingival plaque after 21 days.

Harnacke et al^18^ compared Fones and modified Bass techniques using TQHI and MPI. Those authors found the Fones technique to be superior to the modified Bass method with respect to oral hygiene and gingivitis.

Schlueter et al^45^ compared instructed and non-instructed groups after no instructions, written instructions (leaflet) and verbal instructions supported by a demonstration. The study failed to demonstrate any significant improvement in bacterial plaque scores using TQHI for the modified Bass technique.

Mastroberardino et al^33^ compared horizontal and vertical toothbrushing techniques using the RMNPI. Vertical toothbrushing was more efficient in reducing overall mouth plaque scores and removed more interdental plaque.

In a second study, Harnacke et al^19^ once again compared Fones and modified Bass toothbrushing techniques. The overall reduction of bacterial plaque was slightly greater with the Fones technique (70.3%) compared to the modified Bass technique (77.9%).

In an in vitro study, Jansiriwattana and Teparat-Burana^24^ compared horizontal scrub and modified Bass toothbrushing techniques using O’Leary and PMI indices. The study found no difference among three different toothbrushes with either brushing technique (Table 3).

### Use of the Gingival Index

Out of the thirteen studies selected, only two reported the use of a gingival index. Harnacke et al^18^ reported the use of the papillary bleeding index (PBI) by Saxer and Mühlemann as an indicator of gingivitis. In that study, authors found the Fones technique to have an advantage over the modified Bass method in relation to oral hygiene skills and gingivitis. In their second study, Harnacke et al^19^ used bleeding on probing (BOP) as a variable outcome for gingivitis. In that study, after 12 weeks, instruction in the Fones and the modified Bass techniques failed to show signs of improvement for gingivitis.^19^ None of the other eleven studies reported the use of a gingival index to measure gingivitis (Table 4).

### Assessment of Risk of Bias

**Fig 2:**
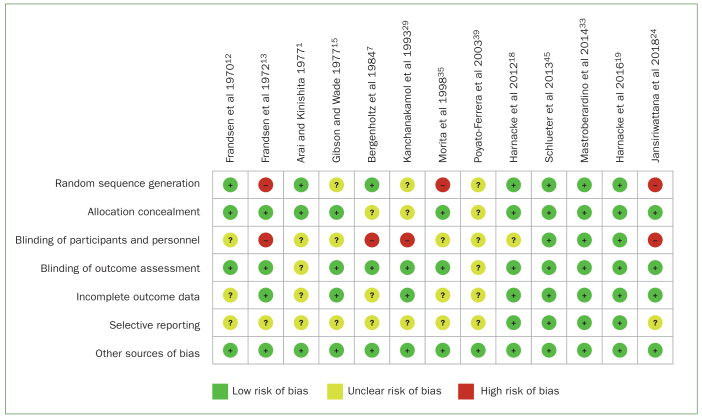
Presentation of risk of bias assessment of included studies.

All five RCT studies exhibit low risk of bias except Arai and Kinoshita,^1^ due to the latter’s vague methodology including unclear blinding of experimenters and participants (Fig 2). The experimental and in vitro studies had at least one high or unclear risk of bias score. In the study by Frandsen et al,^12^ the personnel performing professional brushing were not blinded to the toothbrushing technique to be performed in different participant groups, and the authors failed to mention if there were any dropouts. Further, a detailed protocol was absent, but all expected outcomes were reported. Therefore, it is unclear if this study had selective reporting bias. In a later study, Frandsen et al^13^ divided the participants based on high or low plaque score. Hence, the subjects were not randomly allocated in the research. As in their previous study, they failed to clarify whether the participants were blinded to the name of the technique allocation; furthermore, the study protocol was not available.

Gibson and Wade^15^ performed a cross-over trial where the same participants were utilised as the control and intervention group. The authors did not state how or if the subjects were allocated. Furthermore, it was also not stated whether they were blinded to the toothbrush technique and the study protocol. Therefore, that study is considered to have a high risk of bias, since the participants and/or personnel were not blinded. The same reasons for risk of bias were present in the studies by Bergenholtz et al^7^ and Kanchanakamol et al.^29^

Morita et al^35^ performed randomisation allocations of different conditions to the subjects. However, the process of how this was achieved was not stated explicitly in the article. It is also unclear whether the participants were blinded to the toothbrush technique allocated to them. The cross-over study performed by Poyato-Ferrara et al^39^ was poorly described. As such, this study had an unclear risk of bias in all categories. Jansiriwattana and Teparat-Burana^24^ investigated toothbrushing techniques in vitro. Therefore, there was a high risk of bias as there were no human participants involved. In addition, the dentist who performed the toothbrushing techniques was not blinded, therefore representing a high risk of bias.

## Discussion

### Literature Search

This systematic review highlighted only 13 good-quality studies examining manual toothbrushing effectiveness in relation to toothbrushing techniques. The vast majority of the literature focuses on electric toothbrushing, although manual toothbrushing is still a predominant method of cleaning. We were able to find two systematic reviews in relation to manual toothbrushing. The first review compared efficiency of plaque removal and gingivitis between manual toothbrushes vs powered toothbrushes.^54^ The second systematic review focused on pre- and post-brushing bacterial plaque scores with manual toothbrushes, but mainly focused on bristle-tuft configurations and toothbrushing duration.^47^ The present authors are unaware of other systematic reviews on manual toothbrushing techniques published to date.

In the following, each parameter presented in our results is discussed, as we found it difficult to relate our discussion to similar studies.

### Toothbrushing Techniques

The modified Stillman technique, toothpick technique, vertical and horizontal brushing were only studied once in the thirteen articles included in this systematic review.^1,35,33^ The most common technique examined in six studies was the roll technique.^1,7,12,13,15,29^ The second most common technique compared was the modified Bass, examined by five studies.^18,19,24,39,45^ Ten toothbrushing techniques were assessed in the thirteen publications; the variability made it very difficult to compare. Hence, the validity of the observed results for these techniques is questionable, as they cannot be compared to another study. This presents another limitation in the literature and highlights the need for more scientific studies exploring these specific techniques.

### Methodology of Studies

There are several variations in the design and methodology of these studies. The number of participants varied. Most of the recruited individuals were armed forces personnel, highschool or university students aged between 17 and 30 years old. Only one study recruited university periodontology clinic patients aged 20 to 49. In general, it seems that most of the studies recruited individuals with a highschool and university/armed forces educational background. Toothbrushing instructions and techniques were provided by different approaches, including a combination of the patient’s natural technique, instruction by trained dentist/hygienist/assistant, monitoring by an examiner, computer instructions, pamphlets, videos, and group training. Five studies^7,12,13,33,35^ mentioned seven different training strategies and the help of trained clinicians to brush participant’s teeth. We also found considerable variation within and between studies in terms of toothbrush types and brands. We counted 23 different brands of manual toothbrushes, comparing ten different manual toothbrushing techniques for the 13 selected studies. Once again, in terms of methodology and study design, these 13 studies present too many variations to draw conclusions about a superior toothbrushing technique.

### Comparison of Bacterial Plaque Reduction and Plaque Indices

Only six^1,12,13,15,33,35^ of the thirteen studies reported use of plaque reduction scores. Seven studies did not report percentages of plaque reduction or mean plaque reduction, limiting comparability concerning bacterial plaque removal efficiency of the different manual toothbrushing techniques.

Substantial variation was observed in terms of the different bacterial plaque indices used in these thirteen studies: ten different plaque assessment indices were used. It is also important to acknowledge additional variability in the use of different plaque scoring systems and variations in the number of teeth and scored surfaces per tooth. Two studies implemented Ramfjord teeth, not considering a full-dentition plaque score.^1,15^ Most of the studies included the entire dentition in the plaque score, but the number of surfaces included varied. Three studies scored four surfaces.^7,12,13^ Four studies included six tooth surfaces,^24,35,39,45^ two studies included eight surfaces,^18,29^ and one study^33^ examined eighteen tooth surfaces (Table 4). In light of a recent publication,^10^ the MPI has demonstrated a higher sensitivity compared with the current internationally accepted plaque index, TQHI. The MPI can be used in future studies as it provides a higher reliability and ability to detect a statistically significant difference between two experimental conditions.

Due to the variation of plaque scores reported, reduction of plaque scores and plaque indices, it is hard to conclude that a particular manual toothbrushing technique is more effective than the others for plaque removal.

### Use of a Gingival Index

Out of the thirteen studies, only two (Harnacke et al^18,19^) used true gingivitis assessment tools: PBI and bleeding on probing (BOP). Gingival bleeding on probing is a simple, well-established parameter for demonstrating gingival health and indicating gingivitis.^26,32^ These two studies demonstrated conflicting results, and they only compared two toothbrushing techniques out of the ten included in this review: Fones and modified Bass. In the first study by Harnacke et al,^18^ the Fones technique was superior to the modified Bass for PBI. The second study^19^ failed to find differences in BOP.^19^ To make any consistent conclusion on a particular toothbrushing technique’s ability to reduce gingivitis is extremely difficult, based on conflicting results and the limited number of studies comparing only two toothbrushing techniques.

## Conclusion

Current evidence is inadequate for concluding that one toothbrushing method is more effective than another in plaque removal and reduction of gingival inflammation. Excessive variability in many aspects of the design and methodology of the selected studies make it impossible to reach any conclusion on an ideal manual toothbrushing technique. Experimental randomised controlled trials that follow the CONSORT (consolidated standards of reporting trials) guidelines are required to provide adequate-quality evidence of the relative effectiveness of manual toothbrushing techniques.
